#  A defesa da saúde como um direito de todas as pessoas 

**DOI:** 10.1590/0102-311XPT107923

**Published:** 2023-09-25

**Authors:** Isabel Lopes dos Santos Keppler

**Affiliations:** 1 Universidade Federal de São Paulo, São Paulo, Brasil.

Momento mais propício para discutir o Sistema Único de Saúde (SUS) não há. O sistema de saúde brasileiro passou por um duro teste com a pandemia de COVID-19 e quatro anos de um governo com diversas práticas e discursos que constituíam obstáculos para seu funcionamento.

Mas por que mais um livro sobre SUS? Paulo Capel Narvai, que já produziu em torno de 200 publicações no campo da Saúde Pública, prova como essa temática é inesgotável, não só para atualização do que aconteceu no período mais recente, mas devido à necessidade de voltar décadas atrás, e até mesmo séculos, para compreender como chegamos aonde estamos.

O livro, de forma satisfatória, escapa do compromisso do formato tecnicista e se assemelha a uma conversa com quem o lê. É como se o leitor tivesse passado um café, puxado um banquinho e parado para escutar a experiência acumulada de Narvai nesses mais de 50 anos como intelectual, profissional e militante do SUS.

Um dos fatores que contribuem para essa sensação é o fato de o livro não ter propriamente uma organização convencional de capítulos, como obras clássicas para estudantes e profissionais. Há uma escolha muito autoral e, como em uma conversa, existem várias formas de se começar - e diversos desdobramentos em paralelo.

Ainda que a leitura seja bastante prazerosa e aparentemente despretensiosa, bem como explicitamente se trate de uma visão do autor sobre o SUS, a obra não deixa de ter rigor. O autor transita por importantes referências teóricas, registros factuais históricos e elementos autobiográficos de quem vivenciou os períodos - e participou deles ativamente - antes, durante e depois da reforma sanitária e se posiciona de forma transparente.

Ao longo dos capítulos, Narvai ^1^ defende uma ideia bastante conhecida: o SUS como um direito de todas as pessoas e um dever do Estado. Para isso, recorre à história - do Brasil e da saúde pública - e a debates sobre esse tema. Ao fazer esse percurso, mostra que, embora essa ideia esteja registrada, dessa forma, na *Constituição Federal* de 1988, desde sua origem tal premissa é ameaçada de diversas formas e disputada por diferentes forças políticas e econômicas, nacionais e internacionais.

Do ponto de vista histórico, há um compromisso com a história “vista de baixo”, ou de narrar “*a história que a História não conta*” [ Composição: Deivid Domênico, Tomaz Miranda, Silvio Moreira Filho (Mama), Márcio Bola, Ronie de Oliveira, Danilo Firmino, Manu da Cuíca e Luiz Carlos Máximo. Histórias para Ninar Gente Grande. Samba-enredo da Estação Primeira da Mangueira; 2019], ao romper com o automatismo de contar a história da saúde pública em uma perspectiva eurocêntrica. O autor se dedica a apresentar a interpretação e as formas de cuidado dos povos originários e escravizados. Além disso, não se furta em abordar como o Brasil é constituído e construído sob as marcas de violência, opressão e exploração. O extermínio dos povos originários ocorreu também a partir das doenças trazidas pelos europeus - não apenas no Brasil, mas na América Latina. Depois, houve um longo período de escravização, em que o Brasil foi um dos últimos países a abolir esse sistema e o local que mais recebeu povos escravizados oriundos do continente africano. Muitos foram dizimados desde o caminho, por epidemia, desnutrição e suicídio ^1^.

A saúde pública no Brasil surge como medida para conter uma situação já bastante grave de epidemias e condições insalubres e indignas a qual boa parte da classe trabalhadora era submetida, pós-período colonial. A forma de resolver esses problemas estava muito longe de ser embasada por uma consciência sanitária. Houve a instauração da polícia médica e um modelo “campanhista” muito limitado. Todo esse breve panorama mostra o aspecto “revolucionário” de uma reforma, tal qual a aprovação do SUS, mesmo em uma República com “construção inacabada” como a do Brasil ^1^.

Com relação aos debates, é possível sintetizá-los na polarização entre o que o autor chama de *SUSistas* e *SUScitas*. Os *SUSistas*, são, como explica o autor, aqueles que compreendem a saúde como um direito social fundamental, impassível de ser negado a qualquer ser humano. Os *SUScistas*, por sua vez, defendem ou tomam decisões e posicionamentos tratando a saúde como um bem privado, que se adquire individualmente conforme suas condições. Eles concebem a saúde, ainda, como oposta à doença. Para abordar a polêmica, o autor recorre a polarizações - como Fidel Castro e Barack Obama - e a expressões que, às vezes, são tratadas como sinônimas ou continuidades, mas que têm distinções, tal como atenção primária e atenção básica, necessidades em saúde e necessidades de saúde, Alma-Ata e Astana, entre outras.

Dois temas que são abordados e merecem destaque para refletir sobre o futuro são saúde e desenvolvimento e saúde e democracia. Nesse contexto de emergência climática, é salutar recuperar o processo de industrialização e as estratégias de diferentes governos e pensar como é possível que o progresso industrial e econômico do país não apenas não comprometa o meio ambiente, como também acompanhe o desenvolvimento das condições de vida e de saúde de toda a sociedade. O tópico saúde e democracia, tão presente na 8ª Conferência Nacional de Saúde e na *Constituição Federal*, volta com força nesse contexto recente em que foi tão ameaçado pelo Governo de Jair Messias Bolsonaro e pela pandemia. Vinculada a essa temática, a participação social é abordada também como um elemento central para enfrentar os interesses privatistas e permitir que o projeto da reforma sanitária siga e persista, mesmo nos marcos de um sistema capitalista.

Por fim, o livro indica vários caminhos para refletir e pensar sobre a ideia de que o SUS “não funciona”. A obra mostra, por um lado, que o sistema de saúde sobreviveu a despeito de tantos ataques e que, sim, funciona. Sem o SUS, sem dúvida, estaríamos muito piores. Por outro, deixa em aberto se as dificuldades e os obstáculos persistem apenas por não sermos fortes o suficiente diante da ofensiva neoliberal. Seria possível implementar, dentro do Estado capitalista neoliberal, uma “reforma revolucionária”? Responder a essa pergunta exigiria um aprofundamento maior sobre a concepção de Estado, que não opera na lógica do sensato e racional, mas sim para manutenção do sistema capitalista. Ainda que não adentre esse debate, a conclusão de apostar na participação social e a ideia de uma disputa em aberto entre os interesses mercantis (a destruição do SUS tal qual concebida em sua origem) e os interesses societários (a consolidação dessa “reforma revolucionária”) convocam o leitor a se posicionar em um momento crucial da história.
